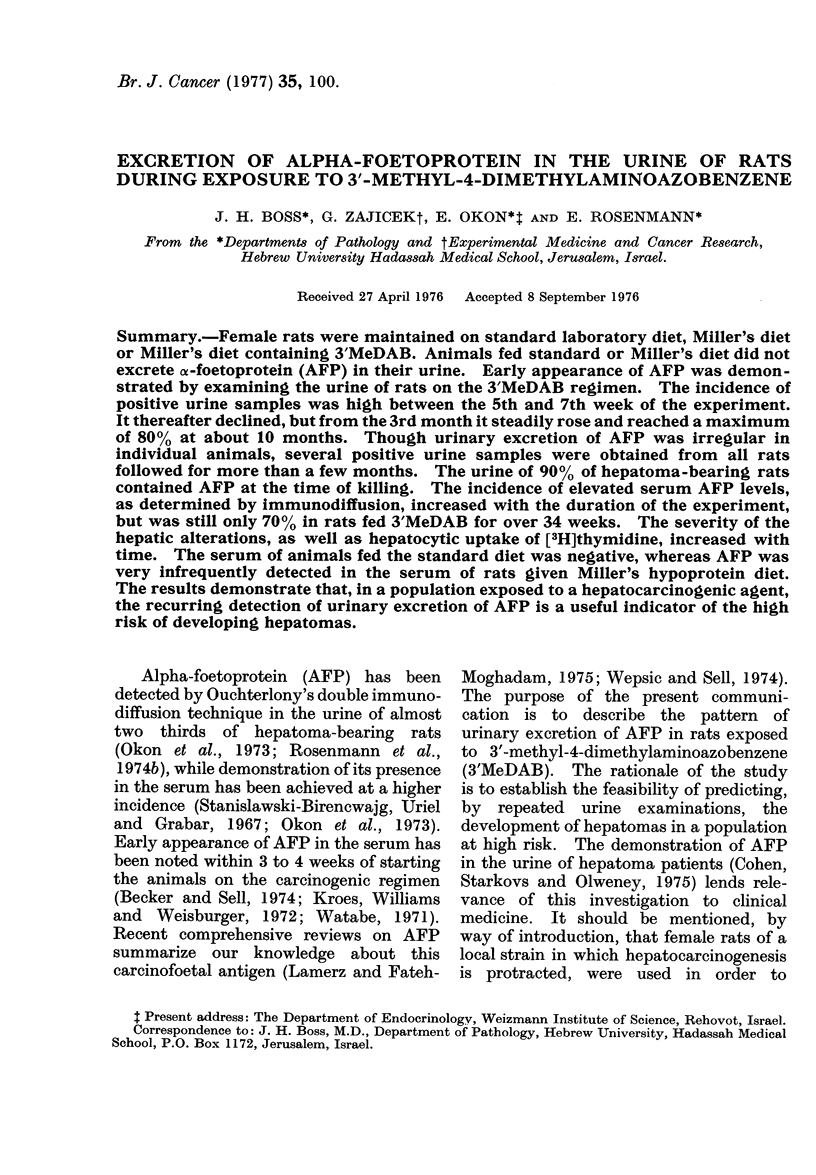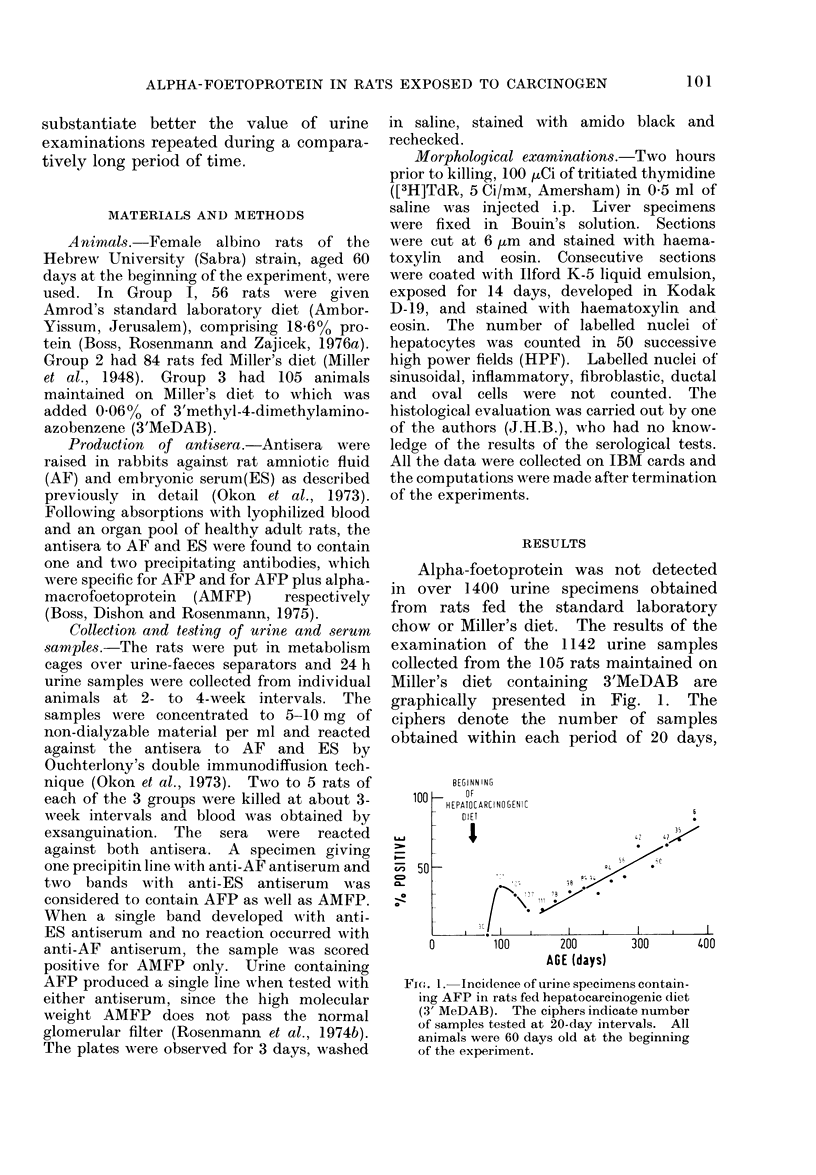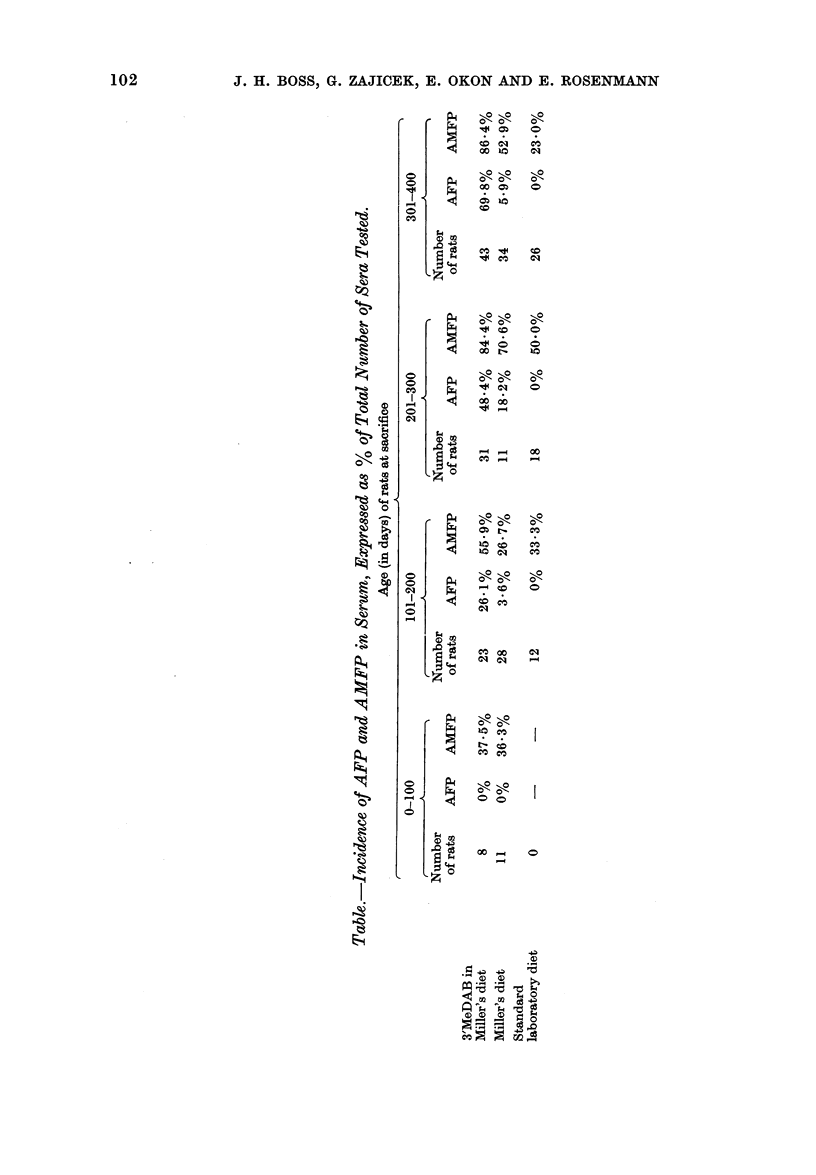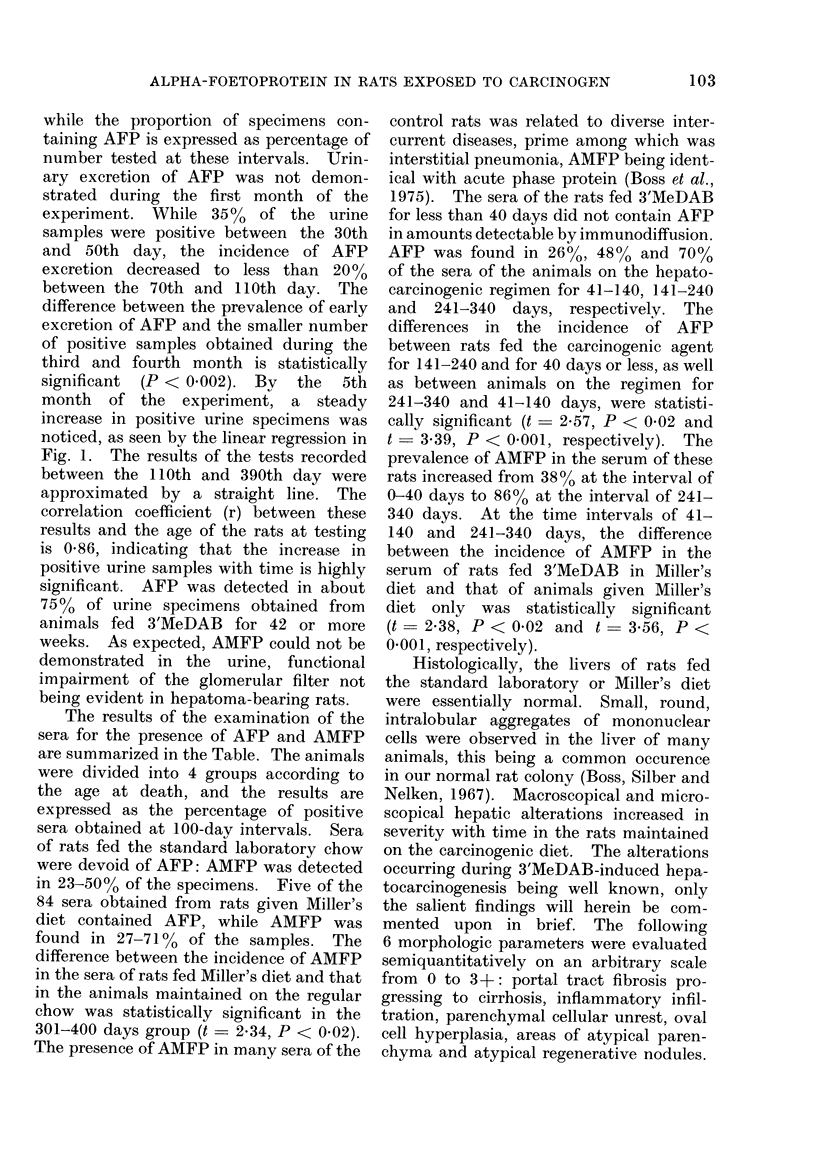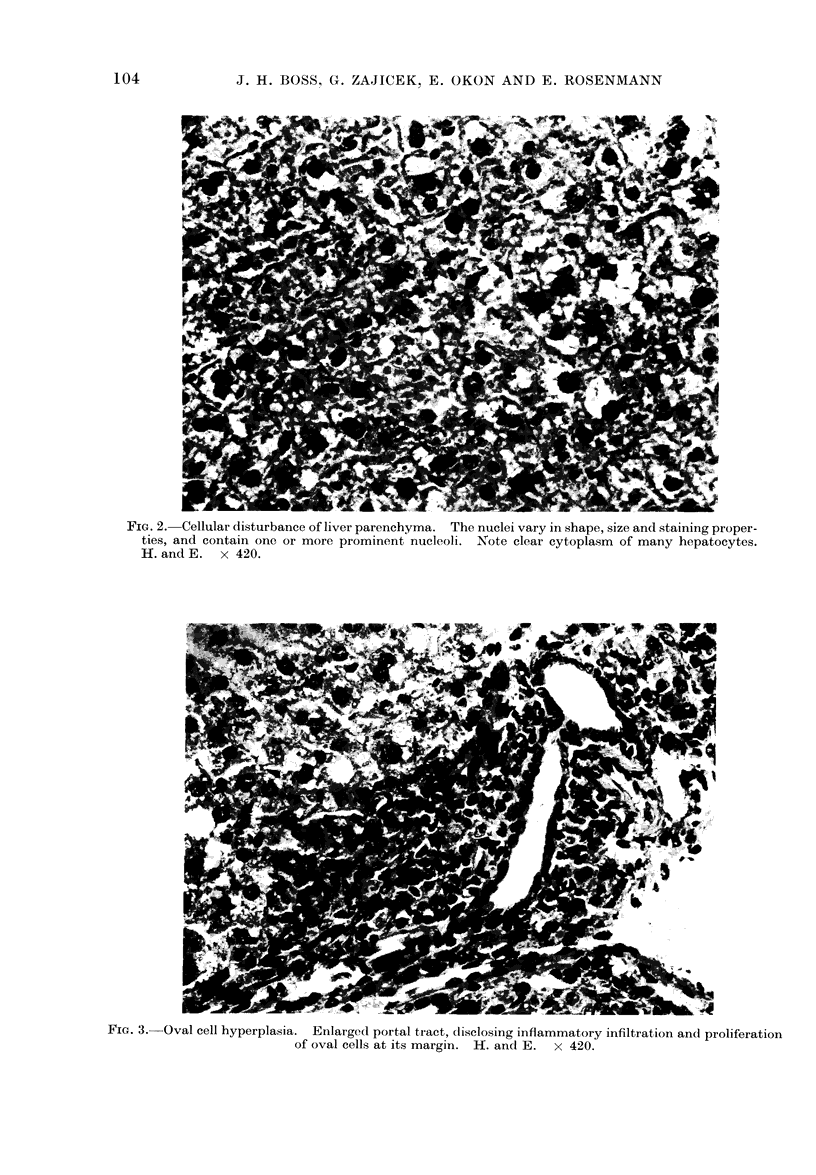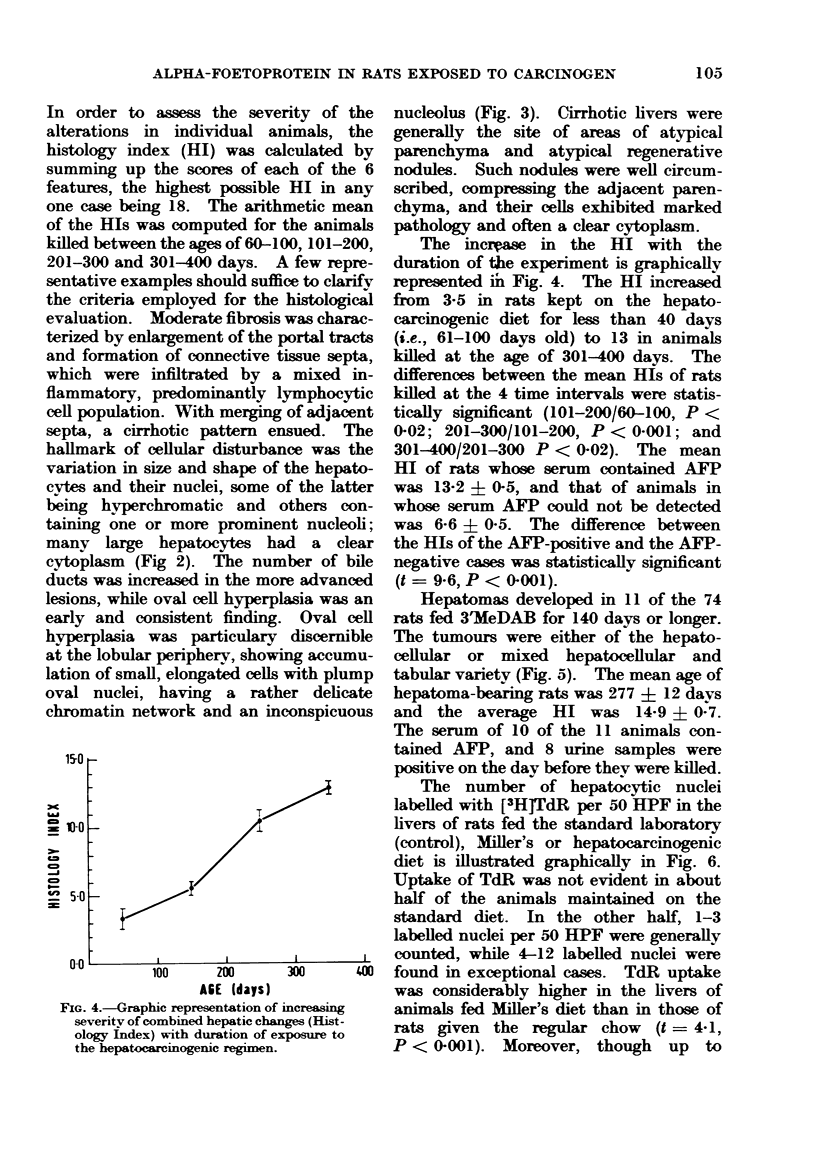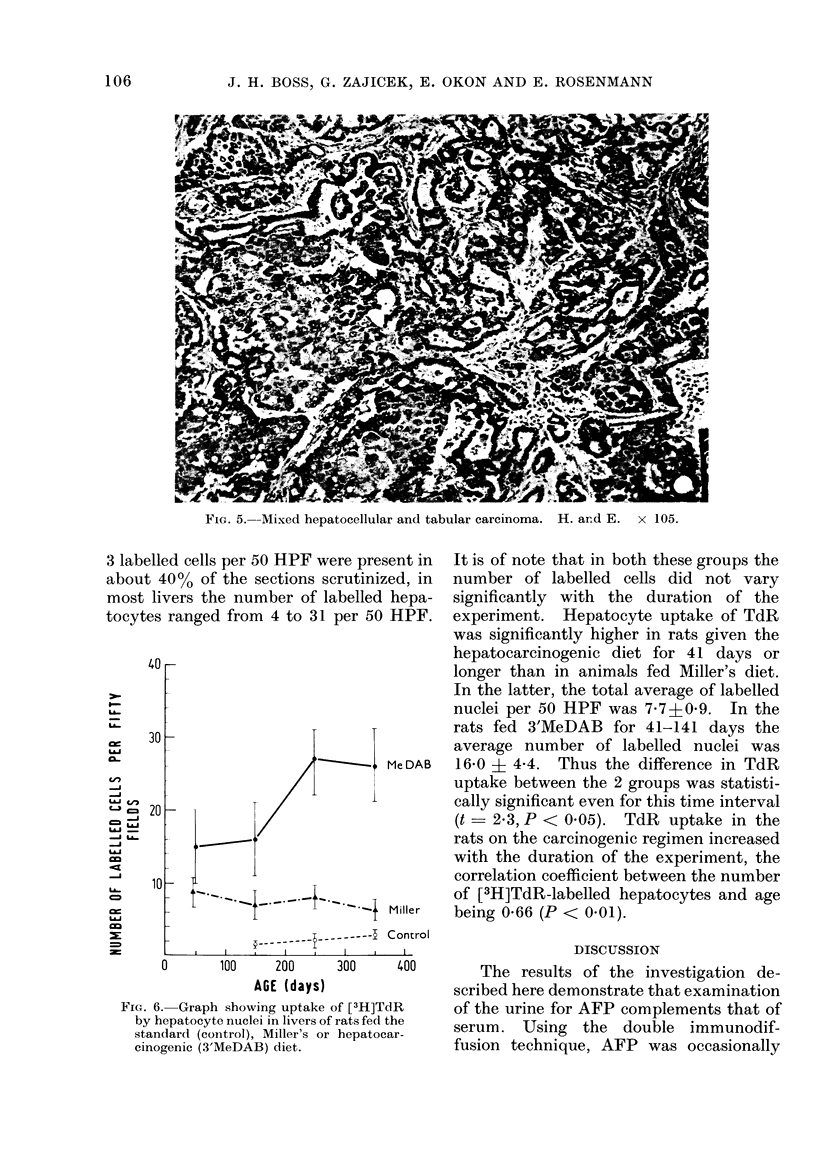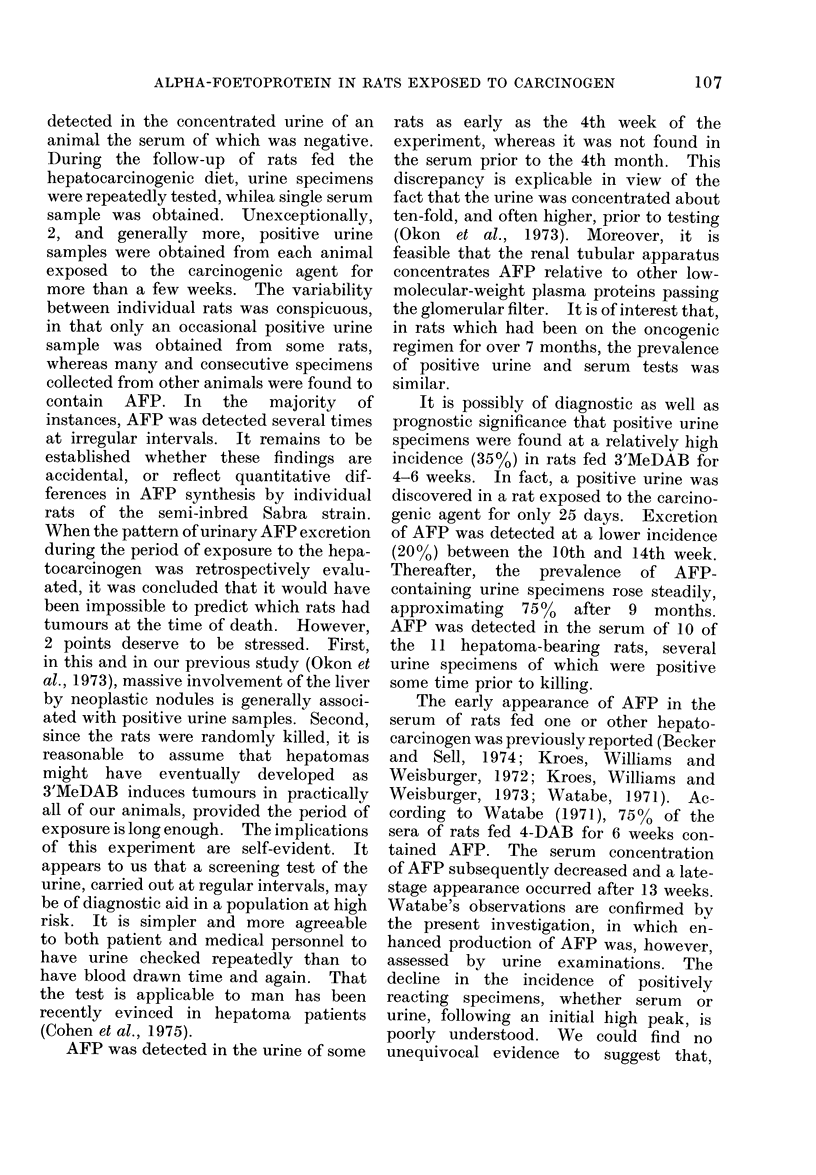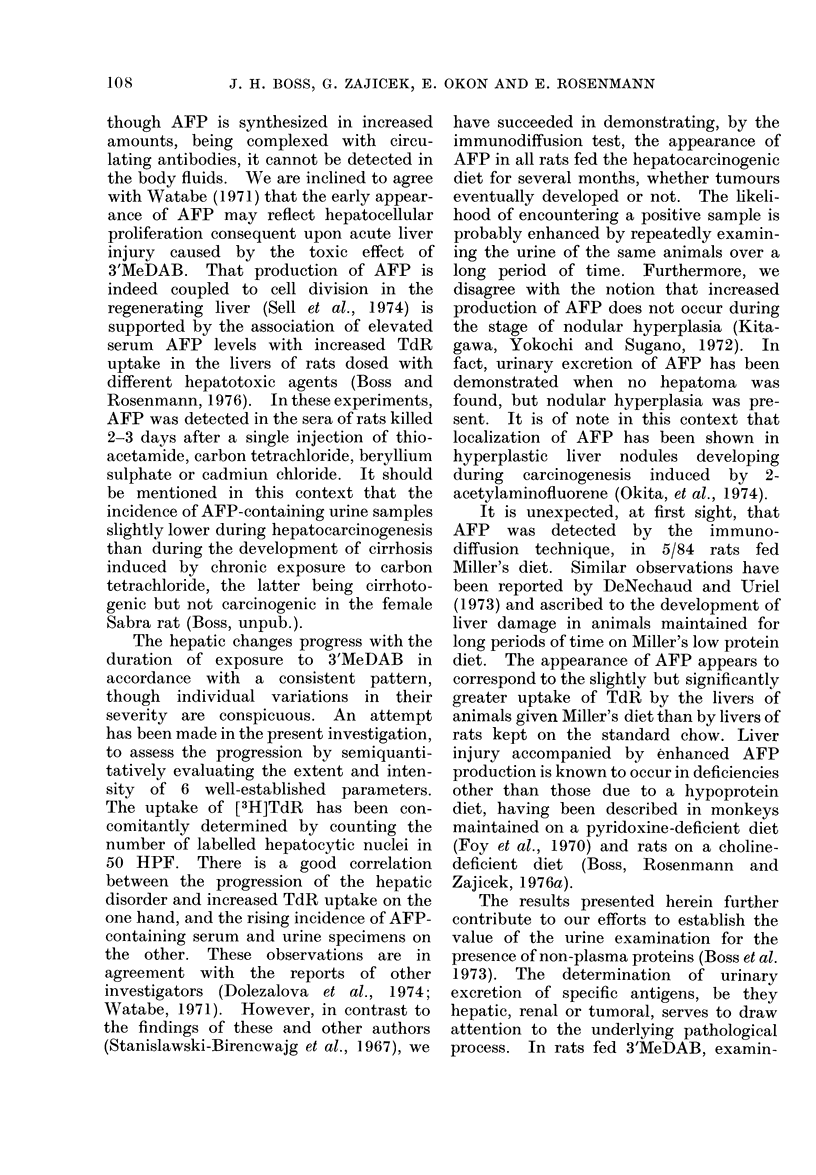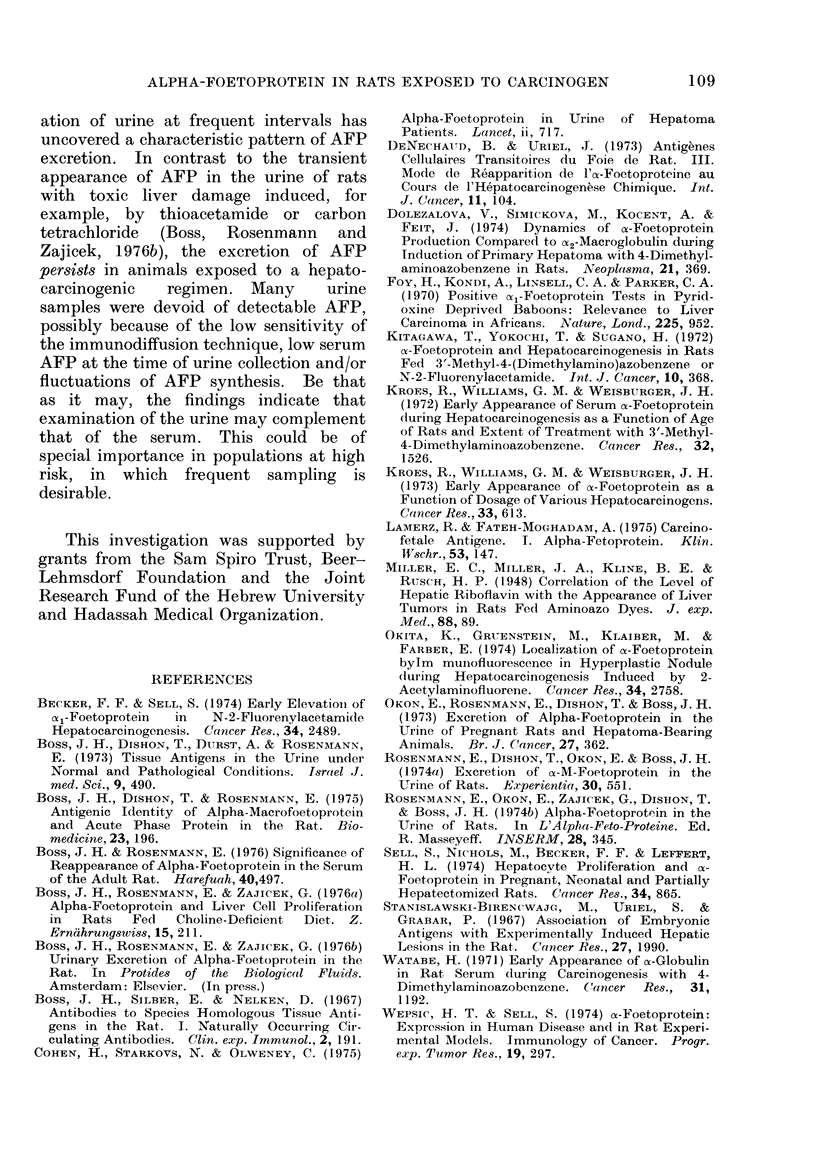# Excretion of alpha-foetoprotein in the urine of rats during exposure to 3'-methyl-4-dimethylaminoazobenzene.

**DOI:** 10.1038/bjc.1977.9

**Published:** 1977-01

**Authors:** J. H. Boss, G. Zajicek, E. Okon, E. Rosenmann

## Abstract

**Images:**


*Br. J. Cancer* (1977) **35**, 100.

### EXCRETION OF ALPHA-FOETOPROTEIN IN THE URINE OF RATS DURING EXPOSURE TO 3′-METHYL-4-DIMETHYLAMINOAZOBENZENE

J. H. BOSS*, G. ZAJICEK†, E. OKON*‡ AND E. ROSENMANN*

*From the *Departments of Pathology and †Experimental Medicine and Cancer Research, Hebrew University Hadassah Medical School, Jerusalem, Israel.*

Received 27 April 1976 Accepted 8 September 1976

**Summary.—Female rats were maintained on standard laboratory diet, Miller's diet or Miller's diet containing 3′MeDAB. Animals fed standard or Miller's diet did not excrete α-foetoprotein (AFP) in their urine. Early appearance of AFP was demonstrated by examining the urine of rats on the 3′MeDAB regimen. The incidence of positive urine samples was high between the 5th and 7th week of the experiment. It thereafter declined, but from the 3rd month it steadily rose and reached a maximum of 80% at about 10 months. Though urinary excretion of AFP was irregular in individual animals, several positive urine samples were obtained from all rats followed for more than a few months. The urine of 90% of hepatoma-bearing rats contained AFP at the time of killing. The incidence of elevated serum AFP levels, as determined by immunodiffusion, increased with the duration of the experiment, but was still only 70% in rats fed 3′MeDAB for over 34 weeks. The severity of the hepatic alterations, as well as hepatocytic uptake of [$^3$H]thymidine, increased with time. The serum of animals fed the standard diet was negative, whereas AFP was very infrequently detected in the serum of rats given Miller's hypoprotein diet. The results demonstrate that, in a population exposed to a hepatocarcinogenic agent, the recurring detection of urinary excretion of AFP is a useful indicator of the high risk of developing hepatomas.**

Alpha-foetoprotein (AFP) has been detected by Ouchterlony's double immunodiffusion technique in the urine of almost two thirds of hepatoma-bearing rats (Okon *et al.*, 1973; Rosenmann *et al.*, 1974*b*), while demonstration of its presence in the serum has been achieved at a higher incidence (Stanislawski-Birencwajg, Uriel and Grabar, 1967; Okon *et al.*, 1973). Early appearance of AFP in the serum has been noted within 3 to 4 weeks of starting the animals on the carcinogenic regimen (Becker and Sell, 1974; Kroes, Williams and Weisburger, 1972; Watabe, 1971). Recent comprehensive reviews on AFP summarize our knowledge about this carcinofoetal antigen (Lamerz and Fateh-Moghadam, 1975; Wepsic and Sell, 1974). The purpose of the present communication is to describe the pattern of urinary excretion of AFP in rats exposed to 3′-methyl-4-dimethylaminoazobenzene (3′MeDAB). The rationale of the study is to establish the feasibility of predicting, by repeated urine examinations, the development of hepatomas in a population at high risk. The demonstration of AFP in the urine of hepatoma patients (Cohen, Starkovs and Olweney, 1975) lends relevance of this investigation to clinical medicine. It should be mentioned, by way of introduction, that female rats of a local strain in which hepatocarcinogenesis is protracted, were used in order to

‡ Present address: The Department of Endocrinology, Weizmann Institute of Science, Rehovot, Israel.
Correspondence to: J. H. Boss, M.D., Department of Pathology, Hebrew University, Hadassah Medical School, P.O. Box 1172, Jerusalem, Israel.

substantiate better the value of urine examinations repeated during a comparatively long period of time.

### MATERIALS AND METHODS

*Animals.*—Female albino rats of the Hebrew University (Sabra) strain, aged 60 days at the beginning of the experiment, were used. In Group I, 56 rats were given Amrod's standard laboratory diet (Ambor-Yissum, Jerusalem), comprising 18·6% protein (Boss, Rosenmann and Zajicek, 1976*a*). Group 2 had 84 rats fed Miller's diet (Miller *et al.*, 1948). Group 3 had 105 animals maintained on Miller's diet to which was added 0·06% of 3′methyl-4-dimethylaminoazobenzene (3′MeDAB).

*Production of antisera.*—Antisera were raised in rabbits against rat amniotic fluid (AF) and embryonic serum(ES) as described previously in detail (Okon *et al.*, 1973). Following absorptions with lyophilized blood and an organ pool of healthy adult rats, the antisera to AF and ES were found to contain one and two precipitating antibodies, which were specific for AFP and for AFP plus alphamacrofoetoprotein (AMFP) respectively (Boss, Dishon and Rosenmann, 1975).

*Collection and testing of urine and serum samples.*—The rats were put in metabolism cages over urine-faeces separators and 24 h urine samples were collected from individual animals at 2- to 4-week intervals. The samples were concentrated to 5–10 mg of non-dialyzable material per ml and reacted against the antisera to AF and ES by Ouchterlony's double immunodiffusion technique (Okon *et al.*, 1973). Two to 5 rats of each of the 3 groups were killed at about 3-week intervals and blood was obtained by exsanguination. The sera were reacted against both antisera. A specimen giving one precipitin line with anti-AF antiserum and two bands with anti-ES antiserum was considered to contain AFP as well as AMFP. When a single band developed with anti-ES antiserum and no reaction occurred with anti-AF antiserum, the sample was scored positive for AMFP only. Urine containing AFP produced a single line when tested with either antiserum, since the high molecular weight AMFP does not pass the normal glomerular filter (Rosenmann *et al.*, 1974*b*). The plates were observed for 3 days, washed in saline, stained with amido black and rechecked.

*Morphological examinations.*—Two hours prior to killing, 100 μCi of tritiated thymidine ([$^3$H]TdR, 5 Ci/mM, Amersham) in 0·5 ml of saline was injected i.p. Liver specimens were fixed in Bouin's solution. Sections were cut at 6 μm and stained with haematoxylin and eosin. Consecutive sections were coated with Ilford K-5 liquid emulsion, exposed for 14 days, developed in Kodak D-19, and stained with haematoxylin and eosin. The number of labelled nuclei of hepatocytes was counted in 50 successive high power fields (HPF). Labelled nuclei of sinusoidal, inflammatory, fibroblastic, ductal and oval cells were not counted. The histological evaluation was carried out by one of the authors (J.H.B.), who had no knowledge of the results of the serological tests. All the data were collected on IBM cards and the computations were made after termination of the experiments.

### RESULTS

Alpha-foetoprotein was not detected in over 1400 urine specimens obtained from rats fed the standard laboratory chow or Miller's diet. The results of the examination of the 1142 urine samples collected from the 105 rats maintained on Miller's diet containing 3′MeDAB are graphically presented in Fig. 1. The ciphers denote the number of samples obtained within each period of 20 days,

Fig. 1.—Incidence of urine specimens containing AFP in rats fed hepatocarcinogenic diet (3′ MeDAB). The ciphers indicate number of samples tested at 20-day intervals. All animals were 60 days old at the beginning of the experiment.

*Table.—Incidence of AFP and AMFP in Serum, Expressed as % of Total Number of Sera Tested.*

| | Age (in days) of rats at sacrifice | | | | | | | | | | | |
|---|---|---|---|---|---|---|---|---|---|---|---|---|
| | 0–100 | | | 101–200 | | | 201–300 | | | 301–400 | | |
| | Number of rats | AFP | AMFP | Number of rats | AFP | AMFP | Number of rats | AFP | AMFP | Number of rats | AFP | AMFP |
| 3′MeDAB in Miller's diet | 8 | 0% | 37·5% | 23 | 26·1% | 55·9% | 31 | 48·4% | 84·4% | 43 | 69·8% | 86·4% |
| Miller's diet | 11 | 0% | 36·3% | 28 | 3·6% | 26·7% | 11 | 18·2% | 70·6% | 34 | 5·9% | 52·9% |
| Standard laboratory diet | 0 | — | — | 12 | 0% | 33·3% | 18 | 0% | 50·0% | 26 | 0% | 23·0% |

while the proportion of specimens containing AFP is expressed as percentage of number tested at these intervals. Urinary excretion of AFP was not demonstrated during the first month of the experiment. While 35% of the urine samples were positive between the 30th and 50th day, the incidence of AFP excretion decreased to less than 20% between the 70th and 110th day. The difference between the prevalence of early excretion of AFP and the smaller number of positive samples obtained during the third and fourth month is statistically significant ($P < 0{\cdot}002$). By the 5th month of the experiment, a steady increase in positive urine specimens was noticed, as seen by the linear regression in Fig. 1. The results of the tests recorded between the 110th and 390th day were approximated by a straight line. The correlation coefficient (r) between these results and the age of the rats at testing is 0·86, indicating that the increase in positive urine samples with time is highly significant. AFP was detected in about 75% of urine specimens obtained from animals fed 3′MeDAB for 42 or more weeks. As expected, AMFP could not be demonstrated in the urine, functional impairment of the glomerular filter not being evident in hepatoma-bearing rats.

The results of the examination of the sera for the presence of AFP and AMFP are summarized in the Table. The animals were divided into 4 groups according to the age at death, and the results are expressed as the percentage of positive sera obtained at 100-day intervals. Sera of rats fed the standard laboratory chow were devoid of AFP: AMFP was detected in 23–50% of the specimens. Five of the 84 sera obtained from rats given Miller's diet contained AFP, while AMFP was found in 27–71% of the samples. The difference between the incidence of AMFP in the sera of rats fed Miller's diet and that in the animals maintained on the regular chow was statistically significant in the 301–400 days group ($t = 2{\cdot}34$, $P < 0{\cdot}02$). The presence of AMFP in many sera of the control rats was related to diverse intercurrent diseases, prime among which was interstitial pneumonia, AMFP being identical with acute phase protein (Boss *et al.*, 1975). The sera of the rats fed 3′MeDAB for less than 40 days did not contain AFP in amounts detectable by immunodiffusion. AFP was found in 26%, 48% and 70% of the sera of the animals on the hepatocarcinogenic regimen for 41–140, 141–240 and 241–340 days, respectively. The differences in the incidence of AFP between rats fed the carcinogenic agent for 141–240 and for 40 days or less, as well as between animals on the regimen for 241–340 and 41–140 days, were statistically significant ($t = 2{\cdot}57$, $P < 0{\cdot}02$ and $t = 3{\cdot}39$, $P < 0{\cdot}001$, respectively). The prevalence of AMFP in the serum of these rats increased from 38% at the interval of 0–40 days to 86% at the interval of 241–340 days. At the time intervals of 41–140 and 241–340 days, the difference between the incidence of AMFP in the serum of rats fed 3′MeDAB in Miller's diet and that of animals given Miller's diet only was statistically significant ($t = 2{\cdot}38$, $P < 0{\cdot}02$ and $t = 3{\cdot}56$, $P < 0{\cdot}001$, respectively).

Histologically, the livers of rats fed the standard laboratory or Miller's diet were essentially normal. Small, round, intralobular aggregates of mononuclear cells were observed in the liver of many animals, this being a common occurence in our normal rat colony (Boss, Silber and Nelken, 1967). Macroscopical and microscopical hepatic alterations increased in severity with time in the rats maintained on the carcinogenic diet. The alterations occurring during 3′MeDAB-induced hepatocarcinogenesis being well known, only the salient findings will herein be commented upon in brief. The following 6 morphologic parameters were evaluated semiquantitatively on an arbitrary scale from 0 to 3+: portal tract fibrosis progressing to cirrhosis, inflammatory infiltration, parenchymal cellular unrest, oval cell hyperplasia, areas of atypical parenchyma and atypical regenerative nodules.

FIG. 2.—Cellular disturbance of liver parenchyma. The nuclei vary in shape, size and staining properties, and contain one or more prominent nucleoli. Note clear cytoplasm of many hepatocytes. H. and E. × 420.

FIG. 3.—Oval cell hyperplasia. Enlarged portal tract, disclosing inflammatory infiltration and proliferation of oval cells at its margin. H. and E. × 420.

In order to assess the severity of the alterations in individual animals, the histology index (HI) was calculated by summing up the scores of each of the 6 features, the highest possible HI in any one case being 18. The arithmetic mean of the HIs was computed for the animals killed between the ages of 60–100, 101–200, 201–300 and 301–400 days. A few representative examples should suffice to clarify the criteria employed for the histological evaluation. Moderate fibrosis was characterized by enlargement of the portal tracts and formation of connective tissue septa, which were infiltrated by a mixed inflammatory, predominantly lymphocytic cell population. With merging of adjacent septa, a cirrhotic pattern ensued. The hallmark of cellular disturbance was the variation in size and shape of the hepatocytes and their nuclei, some of the latter being hyperchromatic and others containing one or more prominent nucleoli; many large hepatocytes had a clear cytoplasm (Fig 2). The number of bile ducts was increased in the more advanced lesions, while oval cell hyperplasia was an early and consistent finding. Oval cell hyperplasia was particulary discernible at the lobular periphery, showing accumulation of small, elongated cells with plump oval nuclei, having a rather delicate chromatin network and an inconspicuous nucleolus (Fig. 3). Cirrhotic livers were generally the site of areas of atypical parenchyma and atypical regenerative nodules. Such nodules were well circumscribed, compressing the adjacent parenchyma, and their cells exhibited marked pathology and often a clear cytoplasm.

FIG. 4.—Graphic representation of increasing severity of combined hepatic changes (Histology Index) with duration of exposure to the hepatocarcinogenic regimen.

The increase in the HI with the duration of the experiment is graphically represented in Fig. 4. The HI increased from 3·5 in rats kept on the hepatocarcinogenic diet for less than 40 days (*i.e.*, 61–100 days old) to 13 in animals killed at the age of 301–400 days. The differences between the mean HIs of rats killed at the 4 time intervals were statistically significant (101–200/60–100, $P < 0{\cdot}02$; 201–300/101–200, $P < 0{\cdot}001$; and 301–400/201–300 $P < 0{\cdot}02$). The mean HI of rats whose serum contained AFP was $13{\cdot}2 \pm 0{\cdot}5$, and that of animals in whose serum AFP could not be detected was $6{\cdot}6 \pm 0{\cdot}5$. The difference between the HIs of the AFP-positive and the AFP-negative cases was statistically significant ($t = 9{\cdot}6, P < 0{\cdot}001$).

Hepatomas developed in 11 of the 74 rats fed 3'MeDAB for 140 days or longer. The tumours were either of the hepatocellular or mixed hepatocellular and tabular variety (Fig. 5). The mean age of hepatoma-bearing rats was $277 \pm 12$ days and the average HI was $14{\cdot}9 \pm 0{\cdot}7$. The serum of 10 of the 11 animals contained AFP, and 8 urine samples were positive on the day before they were killed.

The number of hepatocytic nuclei labelled with [$^3$H]TdR per 50 HPF in the livers of rats fed the standard laboratory (control), Miller's or hepatocarcinogenic diet is illustrated graphically in Fig. 6. Uptake of TdR was not evident in about half of the animals maintained on the standard diet. In the other half, 1–3 labelled nuclei per 50 HPF were generally counted, while 4–12 labelled nuclei were found in exceptional cases. TdR uptake was considerably higher in the livers of animals fed Miller's diet than in those of rats given the regular chow ($t = 4{\cdot}1$, $P < 0{\cdot}001$). Moreover, though up to

FIG. 5.—Mixed hepatocellular and tabular carcinoma. H. and E. × 105.

3 labelled cells per 50 HPF were present in about 40% of the sections scrutinized, in most livers the number of labelled hepatocytes ranged from 4 to 31 per 50 HPF.

FIG. 6.—Graph showing uptake of [$^3$H]TdR by hepatocyte nuclei in livers of rats fed the standard (control), Miller's or hepatocarcinogenic (3'MeDAB) diet.

It is of note that in both these groups the number of labelled cells did not vary significantly with the duration of the experiment. Hepatocyte uptake of TdR was significantly higher in rats given the hepatocarcinogenic diet for 41 days or longer than in animals fed Miller's diet. In the latter, the total average of labelled nuclei per 50 HPF was $7{\cdot}7 \pm 0{\cdot}9$. In the rats fed 3'MeDAB for 41–141 days the average number of labelled nuclei was $16{\cdot}0 \pm 4{\cdot}4$. Thus the difference in TdR uptake between the 2 groups was statistically significant even for this time interval ($t = 2{\cdot}3, P < 0{\cdot}05$). TdR uptake in the rats on the carcinogenic regimen increased with the duration of the experiment, the correlation coefficient between the number of [$^3$H]TdR-labelled hepatocytes and age being $0{\cdot}66$ ($P < 0{\cdot}01$).

### DISCUSSION

The results of the investigation described here demonstrate that examination of the urine for AFP complements that of serum. Using the double immunodiffusion technique, AFP was occasionally detected in the concentrated urine of an animal the serum of which was negative. During the follow-up of rats fed the hepatocarcinogenic diet, urine specimens were repeatedly tested, whilea single serum sample was obtained. Unexceptionally, 2, and generally more, positive urine samples were obtained from each animal exposed to the carcinogenic agent for more than a few weeks. The variability between individual rats was conspicuous, in that only an occasional positive urine sample was obtained from some rats, whereas many and consecutive specimens collected from other animals were found to contain AFP. In the majority of instances, AFP was detected several times at irregular intervals. It remains to be established whether these findings are accidental, or reflect quantitative differences in AFP synthesis by individual rats of the semi-inbred Sabra strain. When the pattern of urinary AFP excretion during the period of exposure to the hepatocarcinogen was retrospectively evaluated, it was concluded that it would have been impossible to predict which rats had tumours at the time of death. However, 2 points deserve to be stressed. First, in this and in our previous study (Okon *et al.*, 1973), massive involvement of the liver by neoplastic nodules is generally associated with positive urine samples. Second, since the rats were randomly killed, it is reasonable to assume that hepatomas might have eventually developed as 3′MeDAB induces tumours in practically all of our animals, provided the period of exposure is long enough. The implications of this experiment are self-evident. It appears to us that a screening test of the urine, carried out at regular intervals, may be of diagnostic aid in a population at high risk. It is simpler and more agreeable to both patient and medical personnel to have urine checked repeatedly than to have blood drawn time and again. That the test is applicable to man has been recently evinced in hepatoma patients (Cohen *et al.*, 1975).

AFP was detected in the urine of some rats as early as the 4th week of the experiment, whereas it was not found in the serum prior to the 4th month. This discrepancy is explicable in view of the fact that the urine was concentrated about ten-fold, and often higher, prior to testing (Okon *et al.*, 1973). Moreover, it is feasible that the renal tubular apparatus concentrates AFP relative to other low-molecular-weight plasma proteins passing the glomerular filter. It is of interest that, in rats which had been on the oncogenic regimen for over 7 months, the prevalence of positive urine and serum tests was similar.

It is possibly of diagnostic as well as prognostic significance that positive urine specimens were found at a relatively high incidence (35%) in rats fed 3′MeDAB for 4–6 weeks. In fact, a positive urine was discovered in a rat exposed to the carcinogenic agent for only 25 days. Excretion of AFP was detected at a lower incidence (20%) between the 10th and 14th week. Thereafter, the prevalence of AFP-containing urine specimens rose steadily, approximating 75% after 9 months. AFP was detected in the serum of 10 of the 11 hepatoma-bearing rats, several urine specimens of which were positive some time prior to killing.

The early appearance of AFP in the serum of rats fed one or other hepatocarcinogen was previously reported (Becker and Sell, 1974; Kroes, Williams and Weisburger, 1972; Kroes, Williams and Weisburger, 1973; Watabe, 1971). According to Watabe (1971), 75% of the sera of rats fed 4-DAB for 6 weeks contained AFP. The serum concentration of AFP subsequently decreased and a late-stage appearance occurred after 13 weeks. Watabe's observations are confirmed by the present investigation, in which enhanced production of AFP was, however, assessed by urine examinations. The decline in the incidence of positively reacting specimens, whether serum or urine, following an initial high peak, is poorly understood. We could find no unequivocal evidence to suggest that, though AFP is synthesized in increased amounts, being complexed with circulating antibodies, it cannot be detected in the body fluids. We are inclined to agree with Watabe (1971) that the early appearance of AFP may reflect hepatocellular proliferation consequent upon acute liver injury caused by the toxic effect of 3′MeDAB. That production of AFP is indeed coupled to cell division in the regenerating liver (Sell *et al.*, 1974) is supported by the association of elevated serum AFP levels with increased TdR uptake in the livers of rats dosed with different hepatotoxic agents (Boss and Rosenmann, 1976). In these experiments, AFP was detected in the sera of rats killed 2–3 days after a single injection of thioacetamide, carbon tetrachloride, beryllium sulphate or cadmiun chloride. It should be mentioned in this context that the incidence of AFP-containing urine samples slightly lower during hepatocarcinogenesis than during the development of cirrhosis induced by chronic exposure to carbon tetrachloride, the latter being cirrhotogenic but not carcinogenic in the female Sabra rat (Boss, unpub.).

The hepatic changes progress with the duration of exposure to 3′MeDAB in accordance with a consistent pattern, though individual variations in their severity are conspicuous. An attempt has been made in the present investigation, to assess the progression by semiquantitatively evaluating the extent and intensity of 6 well-established parameters. The uptake of [$^3$H]TdR has been concomitantly determined by counting the number of labelled hepatocytic nuclei in 50 HPF. There is a good correlation between the progression of the hepatic disorder and increased TdR uptake on the one hand, and the rising incidence of AFP-containing serum and urine specimens on the other. These observations are in agreement with the reports of other investigators (Dolezalova *et al.*, 1974; Watabe, 1971). However, in contrast to the findings of these and other authors (Stanislawski-Birencwajg *et al.*, 1967), we have succeeded in demonstrating, by the immunodiffusion test, the appearance of AFP in all rats fed the hepatocarcinogenic diet for several months, whether tumours eventually developed or not. The likelihood of encountering a positive sample is probably enhanced by repeatedly examining the urine of the same animals over a long period of time. Furthermore, we disagree with the notion that increased production of AFP does not occur during the stage of nodular hyperplasia (Kitagawa, Yokochi and Sugano, 1972). In fact, urinary excretion of AFP has been demonstrated when no hepatoma was found, but nodular hyperplasia was present. It is of note in this context that localization of AFP has been shown in hyperplastic liver nodules developing during carcinogenesis induced by 2-acetylaminofluorene (Okita, *et al.*, 1974).

It is unexpected, at first sight, that AFP was detected by the immunodiffusion technique, in 5/84 rats fed Miller's diet. Similar observations have been reported by DeNechaud and Uriel (1973) and ascribed to the development of liver damage in animals maintained for long periods of time on Miller's low protein diet. The appearance of AFP appears to correspond to the slightly but significantly greater uptake of TdR by the livers of animals given Miller's diet than by livers of rats kept on the standard chow. Liver injury accompanied by enhanced AFP production is known to occur in deficiencies other than those due to a hypoprotein diet, having been described in monkeys maintained on a pyridoxine-deficient diet (Foy *et al.*, 1970) and rats on a choline-deficient diet (Boss, Rosenmann and Zajicek, 1976*a*).

The results presented herein further contribute to our efforts to establish the value of the urine examination for the presence of non-plasma proteins (Boss *et al.* 1973). The determination of urinary excretion of specific antigens, be they hepatic, renal or tumoral, serves to draw attention to the underlying pathological process. In rats fed 3′MeDAB, examination of urine at frequent intervals has uncovered a characteristic pattern of AFP excretion. In contrast to the transient appearance of AFP in the urine of rats with toxic liver damage induced, for example, by thioacetamide or carbon tetrachloride (Boss, Rosenmann and Zajicek, 1976*b*), the excretion of AFP *persists* in animals exposed to a hepatocarcinogenic regimen. Many urine samples were devoid of detectable AFP, possibly because of the low sensitivity of the immunodiffusion technique, low serum AFP at the time of urine collection and/or fluctuations of AFP synthesis. Be that as it may, the findings indicate that examination of the urine may complement that of the serum. This could be of special importance in populations at high risk, in which frequent sampling is desirable.

This investigation was supported by grants from the Sam Spiro Trust, Beer–Lehmsdorf Foundation and the Joint Research Fund of the Hebrew University and Hadassah Medical Organization.

### REFERENCES

BECKER, F. F. & SELL, S. (1974) Early Elevation of $\alpha_1$-Foetoprotein in N-2-Fluorenylacetamide Hepatocarcinogenesis. *Cancer Res.*, **34**, 2489.

BOSS, J. H., DISHON, T., DURST, A. & ROSENMANN, E. (1973) Tissue Antigens in the Urine under Normal and Pathological Conditions. *Israel J. med. Sci.*, **9**, 490.

BOSS, J. H., DISHON, T. & ROSENMANN, E. (1975) Antigenic Identity of Alpha-Macrofoetoprotein and Acute Phase Protein in the Rat. *Biomedicine*, **23**, 196.

BOSS, J. H. & ROSENMANN, E. (1976) Significance of Reappearance of Alpha-Foetoprotein in the Serum of the Adult Rat. *Harefuah*, **40**,497.

BOSS, J. H., ROSENMANN, E. & ZAJICEK, G. (1976*a*) Alpha-Foetoprotein and Liver Cell Proliferation in Rats Fed Choline-Deficient Diet. *Z. Ernährungswiss*, **15**, 211.

BOSS, J. H., ROSENMANN, E. & ZAJICEK, G. (1976*b*) Urinary Excretion of Alpha-Foetoprotein in the Rat. In *Protides of the Biological Fluids*. Amsterdam: Elsevier. (In press.)

BOSS, J. H., SILBER, E. & NELKEN, D. (1967) Antibodies to Species Homologous Tissue Antigens in the Rat. I. Naturally Occurring Circulating Antibodies. *Clin. exp. Immunol.*, **2**, 191.

COHEN, H., STARKOVS, N. & OLWENEY, C. (1975) Alpha-Foetoprotein in Urine of Hepatoma Patients. *Lancet*, ii, 717.

DENECHAUD, B. & URIEL, J. (1973) Antigènes Cellulaires Transitoires du Foie de Rat. III. Mode de Réapparition de l'α-Foetoproteine au Cours de l'Hépatocarcinogenèse Chimique. *Int. J. Cancer*, **11**, 104.

DOLEZALOVA, V., SIMICKOVA, M., KOCENT, A. & FEIT, J. (1974) Dynamics of α-Foetoprotein Production Compared to $\alpha_2$-Macroglobulin during Induction of Primary Hepatoma with 4-Dimethylaminoazobenzene in Rats. *Neoplasma*, **21**, 369.

FOY, H., KONDI, A., LINSELL, C. A. & PARKER, C. A. (1970) Positive $\alpha_1$-Foetoprotein Tests in Pyridoxine Deprived Baboons: Relevance to Liver Carcinoma in Africans. *Nature, Lond.*, **225**, 952.

KITAGAWA, T., YOKOCHI, T. & SUGANO, H. (1972) α-Foetoprotein and Hepatocarcinogenesis in Rats Fed 3′-Methyl-4-(Dimethylamino)azobenzene or N-2-Fluorenylacetamide. *Int. J. Cancer*, **10**, 368.

KROES, R., WILLIAMS, G. M. & WEISBURGER, J. H. (1972) Early Appearance of Serum α-Foetoprotein during Hepatocarcinogenesis as a Function of Age of Rats and Extent of Treatment with 3′-Methyl-4-Dimethylaminoazobenzene. *Cancer Res.*, **32**, 1526.

KROES, R., WILLIAMS, G. M. & WEISBURGER, J. H. (1973) Early Appearance of α-Foetoprotein as a Function of Dosage of Various Hepatocarcinogens. *Cancer Res.*, **33**, 613.

LAMERZ, R. & FATEH-MOGHADAM, A. (1975) Carcinofetale Antigene. I. Alpha-Fetoprotein. *Klin. Wschr.*, **53**, 147.

MILLER, E. C., MILLER, J. A., KLINE, B. E. & RUSCH, H. P. (1948) Correlation of the Level of Hepatic Riboflavin with the Appearance of Liver Tumors in Rats Fed Aminoazo Dyes. *J. exp. Med.*, **88**, 89.

OKITA, K., GRUENSTEIN, M., KLAIBER, M. & FARBER, E. (1974) Localization of α-Foetoprotein byIm munofluorescence in Hyperplastic Nodule during Hepatocarcinogenesis Induced by 2-Acetylaminofluorene. *Cancer Res.*, **34**, 2758.

OKON, E., ROSENMANN, E., DISHON, T. & BOSS, J. H. (1973) Excretion of Alpha-Foetoprotein in the Urine of Pregnant Rats and Hepatoma-Bearing Animals. *Br. J. Cancer*, **27**, 362.

ROSENMANN, E., DISHON, T., OKON, E. & BOSS, J. H. (1974*a*) Excretion of α-M-Foetoprotein in the Urine of Rats. *Experientia*, **30**, 551.

ROSENMANN, E., OKON, E., ZAJICEK, G., DISHON, T. & BOSS, J. H. (1974*b*) Alpha-Foetoprotein in the Urine of Rats. In *L'Alpha-Feto-Proteine*. Ed. R. Masseyeff. *INSERM*, **28**, 345.

SELL, S., NICHOLS, M., BECKER, F. F. & LEFFERT, H. L. (1974) Hepatocyte Proliferation and α-Foetoprotein in Pregnant, Neonatal and Partially Hepatectomized Rats. *Cancer Res.*, **34**, 865.

STANISLAWSKI-BIRENCWAJG, M., URIEL, S. & GRABAR, P. (1967) Association of Embryonic Antigens with Experimentally Induced Hepatic Lesions in the Rat. *Cancer Res.*, **27**, 1990.

WATABE, H. (1971) Early Appearance of α-Globulin in Rat Serum during Carcinogenesis with 4-Dimethylaminoazobenzene. *Cancer Res.*, **31**, 1192.

WEPSIC, H. T. & SELL, S. (1974) α-Foetoprotein: Expression in Human Disease and in Rat Experimental Models. Immunology of Cancer. *Progr. exp. Tumor Res.*, **19**, 297.